# Spatio-Temporal Characterization Analysis and Water Quality Assessment of the South-to-North Water Diversion Project of China

**DOI:** 10.3390/ijerph16122227

**Published:** 2019-06-24

**Authors:** Xizhi Nong, Dongguo Shao, Yi Xiao, Hua Zhong

**Affiliations:** State Key Laboratory of Water Resources and Hydropower Engineering Science, Wuhan University, Wuhan 430072, China; nongxizhi@whu.edu.cn (X.N.); zhonghua21cn@126.com (H.Z.)

**Keywords:** water quality assessment, correlation analysis, principal component analysis, water quality index, south-to-north water diversion project of China

## Abstract

In this article, a data matrix of 20 indicators (6960 observations) was obtained from 29 water quality monitoring stations of the Middle Route (MR) of the South-to-North Water Diversion Project of China (SNWDPC). Multivariate statistical techniques including analysis of variance (ANOVA), correlation analysis (CA), and principal component analysis (PCA) were applied to understand and identify the interrelationships between the different indicators and the most contributive sources of anthropogenic and natural impacts on water quality. The water quality index (WQI) was used to assess the classification and variation of water quality. The distributions of the indicators revealed that six heavy-metal indicators including arsenic (As), mercury (Hg), cadmium (Cd), chromium (Cr), selenium (Se), and lead (Pb) were within the Class I standard, while the As, Pb, and Cd displayed spatial variation. Moreover, some physicochemical indicators such as dissolved oxygen, 5-day biochemical oxygen demand (as BOD_5_), and total phosphorus (TP) had spatio-temporal variability. The correlation analysis result demonstrated that As, Hg, Cd, Cr, Se, Pb, copper (Cu), and zinc (Zn) had high correlation coefficients. The PCA result extracted three principal components (PC) accounting for 82.67% of the total variance, while the first PC was indicative of the mixed sources of anthropogenic and natural contributions, the second and the third PCs were mainly controlled by human activities and natural sources, respectively. The calculation results of the WQI showed an excellent water quality of the MR of the SNWDPC where the values of the stations ranged from 10.49 to 17.93, while Hg was the key indicator to determine the WQI > 20 of six stations, which indicated that the Hg can be the main potential threat to water quality and human health in this project. The result suggests that special attention should be paid to the monitoring of Hg, and the investigation and supervision within the areas of high-density human activities in this project should be taken to control the impacts of urban and industrial production and risk sources on water quality.

## 1. Introduction

With the rapid economic development and increase in human populations, water quality deterioration has become a crucial global issue that can lead to serious public health hazards, biodiversity destruction, and aquatic environmental problems [1]. Much long-term research on different water quality problems, such as industrial wastewater discharge [2], heavy metal pollution [3], and land management influences [4], have been conducted from a regional to a global scale [5]. The spatial-temporal variations and trends of water quality can reflect the geographical differences in the sources of pollution and the types of human activities [6], and previous studies have reported that the water quality in different water bodies was easily affected by a wide range of factors, including climate change [7], natural sources [8], and anthropogenic activities particularly mining [9], sewage discharge [10], and agricultural and urban industrial pollutants [11], for instance, some heavy metal pollution was primarily from the use of agricultural pesticides and fertilizers [12], metal refining [13], and vehicle exhaust emissions [14], etc. In addition, water pollution also related to natural processes, ref. [15] such as mineral weathering [16], pedogenesis [17], and dust fall [18], while some synergistic effects can cause water quality changes, such as a dissolved oxygen deficit and eutrophication, that easily lead to algal blooms [19]. Nowadays, the threats of multiple factors to the water environment have raised new challenges for the protection and management of water quality and quantity [20].

In order to alleviate the impacts of water shortage and water deterioration on human society, numerous water diversion projects have been built in the past few decades. For example, the Chinese government has established the world’s largest inter-basin water diversion project, the Middle Route (MR) of the South-to-North Water Diversion Project of China (SNWDPC), to deliver water annually to four provinces of North China since 2014 [21]. Due to the long-distance open channel delivery and complex water regulation, the water quality of this project can be affected by various environmental factors and has certain spatial and temporal variation characteristics [22]. However, the spatio-temporal changes of water quality indicators and their interrelations in this project have seldom been researched and analyzed. Additionally, the current evaluation method cannot fully reflect the actual classification and variation of water quality, resulting in the loss of abundant information contained in massive raw data [23]. Hence, it is extremely important to understand the spatio-temporal characterization and the main sources of impacts on water quality and evaluate the water quality in a more effective manner for the water resource management and public health protection of the MR of the SNWDPC.

The comprehensive application of multivariate statistical techniques such as analysis of variance (ANOVA), correlation analysis (CA), and principal component analysis (PCA) can improve the efficiency of data pre-processing and have been widely used in various cases of environmental studies [24,25,26], especially to analyze and reduce the dimensionality of large datasets, which can retain rich information and mine the relationships among different types of data [27]. The water quality index (WQI) method is a practical and efficient tool that can easily and rapidly evaluate water quality from vast quantities of variables or original data, and has been applied in the assessment of many water bodies [28,29].

The objectives of this study were to (1) analyze the spatio-temporal characterization of the water quality indicators of the MR of the SNWDPC, (2) identify and understand the most contributive natural and/or anthropogenic sources of these indicators, and (3) assess the water quality classification and variation using the WQI method. Ultimately, this research will help to develop water quality management strategies for the respective departments and provide a management reference for other water diversion projects and water environmental research.

## 2. Materials and Methods

### 2.1. Background and Case Study

The MR of the SNWDPC originates in the Danjiangkou Reservoir of the Hubei Province and ends in the Tuancheng Lake, Beijing, and delivers 9.5 billion m^3^ water to four provinces of North China every year [30]. The main canal of the MR of the SNWDPC is 1273.4 km and uses an open channel for water conveyance, accompanying various cross-construction, drainage construction, and bridges [31]. The design flow of the canal head is 350 m^3^/s and provides domestic, industrial, and agricultural water to 19 large cities (with populations over one million), medium-sized cities (with populations between 500,000–1 million), and more than 100 county towns in North China, including Beijing, Tianjin Municipality, the Henan Province, and the Hebei Province, with 61 million people in total [32]. The project crosses the subtropical monsoon climate zone to the temperate monsoon climate zone, with an annual mean air temperature of 15.9–21.2 °C and an annual mean rainfall of 703.6–1173.4 mm [33].

**Figure 1:**
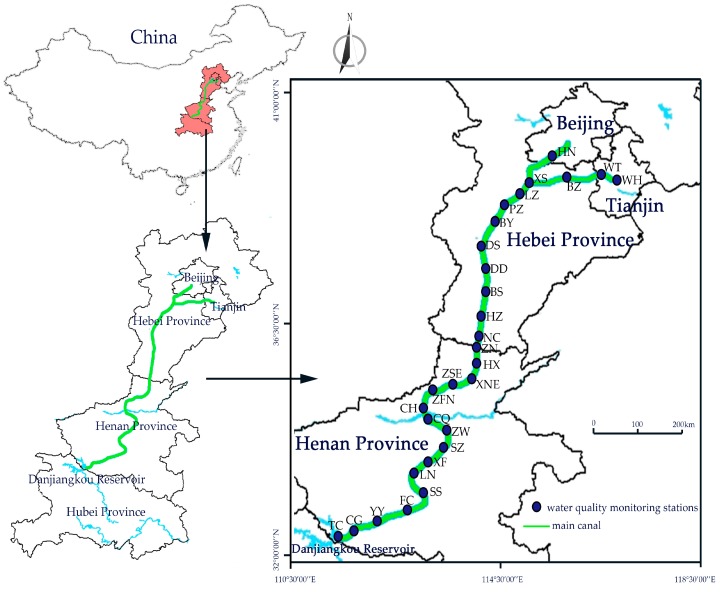
Locations of the 29 water quality monitoring stations of the MR of the SNWDPC.

In order to monitor and control the water quality of the MR of the SNWDPC, the Middle Route Construction Management Bureau of the South-to-North Water Diversion Project has installed 29 fixed water quality monitoring stations along the main canal to test and collect water quality data each month since 2015. There are 16 stations in the Henan Province, 10 in the Hebei Province, two in Tianjin Municipality, and one in Beijing Capital (Table 1 and Figure 1).

### 2.2. Data Collection and Preparation

As the safety of the water quality of the MR of the SNWDPC is highly related to important economic and political issues in China, the government has never allowed a third-party research team to carry out water quality sampling and testing work, and the water sampling campaigns have only ever been conducted by the South-to-North Water Diversion Project Construction Committee Office of the State Council of China. On the basis of compliance with confidentiality agreements and the government’s permission, the data were obtained by the research team of the State Key Laboratory of Water Resources and Hydropower Engineering Science, Wuhan University. The investigation was supported by the Middle Route Construction Management Bureau of the South-to-North Water Diversion Project. Twenty indicators that were set as the basic items in the Environmental Quality Standards for Surface Water, China (No. GB3838-2002) were obtained from 29 water quality monitoring stations per month from December 2015 to November 2016, with 6,960 observations in total. All of the water samples were collected under sunny or cloudy weather conditions to minimize the effects of rainfall or other extreme weather. These indicators included mercury (Hg), lead (Pb), arsenic (As), cadmium (Cd), selenium (Se), chromium (Cr), 5-day biochemical oxygen demand (BOD_5_), pH, dissolved oxygen (DO), permanganate index (as PI), ammonia nitrogen (as NH_3_^–^ N), fecal coliform (as FC), total phosphorus (as TP), total nitrogen (as TN), copper (Cu), zinc (Zn), petroleum, sulfate (as SO_4_^2−^), fluoride (as F^−^), and water temperature (as WT).

Some indicators, such as the water temperature, pH, and dissolved oxygen, were measured in situ by using a multi-parameter probe with a Hydrolab Datasonde 5 sensor. All of the water samples were kept in cold storage and directly transferred to the laboratory at each station using a cooler filled with ice. The concentrations of Hg, Pb, As, Cd, Se, Cr, Cu, and Zn were measured using the inductively-coupled plasma atomic emission spectrometry method (ICP-AES) with an Agilent 7900 inductively coupled plasma-mass spectrometry (ICP-MS; Agilent Technologies Inc.; Santa Clara, Calif., USA). The BOD_5_ was measured as the variation in the oxygen concentration from the beginning to the fifth-day in bottles after incubation under 20 ± 1 °C conditions and assayed by the Winkler method. The concentrations of the permanganate index were tested by titrimetric analysis (potassium permanganate oxidation) using a titration assembly. The contents of TP and TN were measured using a combined potassium persulfate oxidation method. The concentrations of NH_3_^–^ N were analyzed spectrophotometrically using the nesslerization method with a UV spectrophotometer (UV 2450). The petroleum concentrations were determined by the infrared spectrophotometry. The concentrations of SO_4_^2−^ and F^−^ were determined using an ion chromatograph with ion chromatography system. The contents of fecal coliform were determined by the filter membrane method.

The periods of four seasons in this study were defined as spring (March to May), summer (June to August), autumn (September to November), and winter (January, February, and December). Since there were some indicators that were not recorded with specific concentrations, but recorded as “<a” or “≤a”, in order to reflect the difference between these two data records without specific concentrations and to facilitate data calculation and analysis, in this study, when the recorded concentration of an indicator at a certain station was “<a” or not detected, it means that the concentration of this indicator at that station was “0” at a certain moment, and when the recorded concentration of an indicator at a certain station was “≤a”, it means that the concentration of this indicator at that station was “a” at a certain moment.

### 2.3. Multivariate Statistical Methods

One-way analysis of variance (one-way ANOVA) was used to classify and test the seasonal significance of water quality indicators at different stations (*P* < 0.05, least significant difference) [34]. The Pearson coefficient was applied to study and interpret the relationships and interactions among the different indicators. A correlation coefficient close to 1 or −1 indicates the strongest positive or negative correlation between two variables, while a value close to 0 indicates that there is no linear relationship between them at a statistically significant level with *P* < 0.05 and/or *P* < 0.01. Due to the variety of water quality indicators and large amount of raw data, and the fact that there are often different degrees of connection and relationships among different indicators, it is easy to cause information overlaps. The principal component analysis (PCA) is a multivariate statistical technique used to reduce the dimension of raw data by eliminating overlapping information through projection methods, under the principle of ensuring the minimum loss of original information in the system [3,35,36] and to study the underlying structure of the dataset and further understand the relationships among those indicators and the sources of influence on them [37]. In this study, the principal components were retained with an eigenvalue > 1 [38]. The raw data were mainly processed using SPSS 23.0 for Windows. The KMO (Kaiser–Meyer–Olkin) test and Bartlett’s test of sphericity were used to check the data normality for further analysis. The KMO index was applied to compare the correlations between different variables and those of the partial correlations, i.e., the closer the KMO index is to 1, the more suitable the principal component analysis of the variable. The Bartlett’s test of sphericity was used to test the null hypothesis that the intercorrelation came from the groups with unrelated variables.

### 2.4. Water Quality Index

The WQI method has been widely used to evaluate and monitor water quality in various environmental research. The calculation of WQI has been improved and modified by numerous scholars using different mathematical methods under different actual situations and variables. The WQI in this study was based on the recommendation of the Water Environment Monitoring Research Center of the Ministry of Water Resources, China. The classification and the threshold of each grade of different water quality indicators were evaluated in line with the Environmental Quality Standards for Surface Water, China (No. GB3838-2002). The standards divide each water quality item into Class I to V, presenting excellent (I), good (II), medium (III), poor (IV), or very poor (V) standards of water quality (Table 2) [39,40,41]. The 20 water quality indicators were classified into three types, including the Toxic Metals Group, the Easily Treated Indicators Group, and the Other Indicators Group. The Toxic Metals Group in this article included As, Hg, Pb, Cd, Cr, and Se, which had low concentrations in the water of this project. However, the Toxic Metals Group had characteristics of toxicity, persistence, and bioaccumulation that would seriously threaten human life and health, cause water pollution, and would be difficult to treat and purify once exceeding a certain standard. The Easily Treated Indicators Group included the pH, DO, NH_3_^–^ N, FC, and BOD_5_, which are important biochemical indicators and can be easily treated by sewage treatment plants, according to the actual Chinese situation. The Other Indicators Group included the TP, TN, Cu, Zn, petroleum, SO_4_^2−^, F^−^, and water temperature. The WQI values of each station in different seasons were obtained by the steps as follows.

(1) The WQI values of each water quality indicator (as $I_{i}$) were calculated based on Equation (1):

(1) $$I_{i}\;=\;\{\begin{array}{c} \frac{\left(C_{i}\;-\;C_{i,k}\right)}{\left(C_{i,k+n}\;-\;C_{i,k}\right)}\;\times \;20n+I_{i,k},\;C_{i}\in [C_{i,k},C_{i,k+1})\\ \frac{C_{i}}{C_{i,k+n}}\;\times \;20n,\;\;C_{i}\in [0,C_{i,k}) \end{array}$$

In Equation (1), $C_{i}$ is the real concentration of the *i*-th water quality indicator, $C_{i,k}$ and $C_{i,k+n}$ are the standard concentrations of the grade *K* and grad *K* + *n* of the *i*-th water quality indicator respectively, $I_{i,k}$ is the *K* value of the classification item of assessment, and n is the number of the same standard threshold, *n* = 1 when there is no equal standard value.

For pH, when pH ∈ [6,9], $I_{i}$ = 0, otherwise $I_{i}\;$= 100. For undetected indicators, $I_{i}\;$= 0. For the indicator SO_4_^2−^ that only has one standard value, its WQI was calculated based on Equation (2):

(2) $$I_{i}\;=\;\frac{C_{i}}{C_{i,3}}\;\times \;60$$

(2) The grouped WQI values (CI) were calculated for the three groups, where CI(1) for the Toxic Metals Group was based on Equation (3), while CI(2) and CI(3) for the Easily Treated Indicators Group and the Other Indicators Group were based on Equation (4), respectively.

(3) $$CI\;=\;\max\left(I_{i}\right)$$

(4) $$CI\;=\;\frac{\sum_{i=1}^{n}C_{i}P_{i}}{\sum_{i=1}^{n}P_{i}}$$

In Equation (4), *n* is the total number of indicators, $P_{i}$ is the weight of the *i*-th water quality indicator, which presents the importance of the *i*-th indicator for the water use of aquatic life/humans, and the relative values of weight were selected according to previous studies [42,43].

(3) The final WQI for a station in a specific time was calculated based on Equation (5):

(5) $$WQI_{obj}\;=\;\max\left(CI\left(1\right),CI\left(2\right),CI\left(3\right)\right)$$

The water quality classifications were made based on WQI values. Wu evaluated the water quality in Lake Poyang, China, and the water quality was classified from Class I to V, which corresponded to excellent, good, moderate, poor, and bad water states, respectively [40]. Wu discussed the key water quality indicators in Lake Taihu Basin, China, by using the minimum WQI method, which was developed based on a stepwise linear regression analysis, and the water quality was classified into five grades based on the WQI scores [41]. Many international water bodies have also been assessed for water quality by comprehensive multivariate statistical methods [44]. Considering the classification methods of various water quality grades, the actual situation of the Chinese water quality assessment standards, and the water quality management of this project, the classification of WQI values in this study were divided into five grades from 0 to 100, corresponding to the water quality levels from excellent to very poor, as seen in Table 2 and Table 3.

## 3. Results and Discussion

### 3.1. Spatio-temporal Characterization of Water Quality Indicators

#### 3.1.1. Toxic Metals

**Figure 2:**
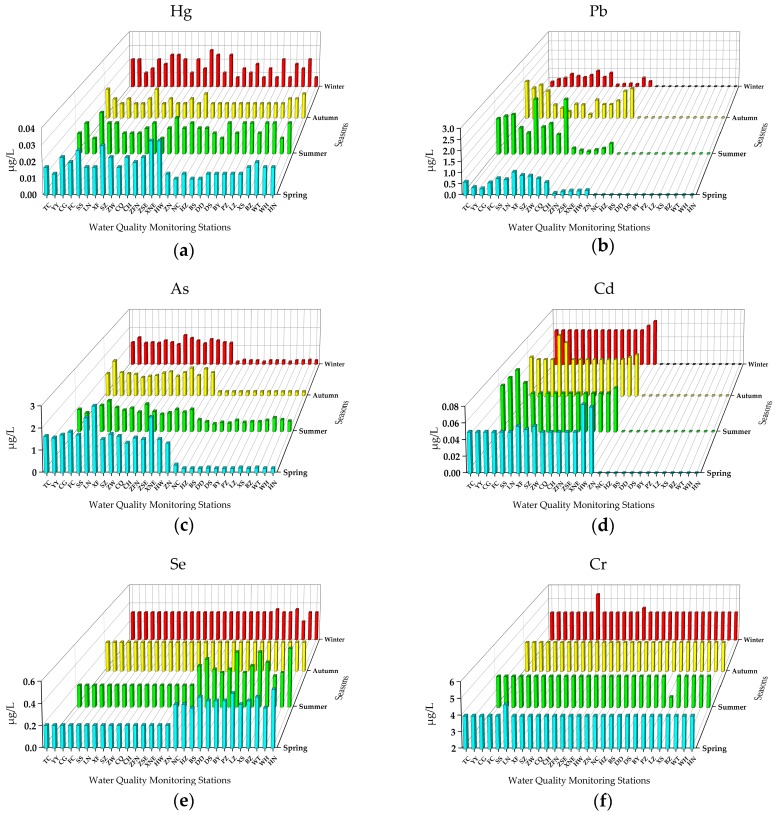
The concentrations of the six metal indicators at 29 stations in the MR of the SNWDPC.

The concentrations of six toxic metal indicators of 29 stations are shown in Figure 2 and Table 4. All toxic metals indicators met the Class I of Standard (No. GB3838-2002) across the four seasons. Since most of the real concentrations of six toxic metals in the MR of the SNWDPC were very low, there were some undetected cases. The Hg and Pb of the 29 stations exhibited significant differences among the four seasons (one-way ANOVA, *P* < 0.05), while As, Cd, Cr, and Se did not (one-way ANOVA, *P* > 0.05).

The annual mean concentration of Hg was 0.0156 μg/L, and the seasonal concentrations ranged from 0.0120 μg/L (summer) to 0.0182 μg/L (spring). The maximum detected Hg appeared in two adjacent stations, XNE and HW (spring, 0.033 μg/L), which indicates that some external Hg sources input to these stations during spring. The Hg concentrations exhibited volatility from the southern to northern stations in four seasons, and there was no obvious spatial distribution rule (Figure 2a). The maximum annual mean concentration of Hg was at the XNE station (0.0225 μg/L), while all of the Hg contents at the 29 stations were lower than the threshold of Class I (0.05 μg/L). Compared with the concentrations of Hg in the Danjiangkou Reservoir, the contents of Hg at some stations were distinctly different to that in the headwater TC station (Table 4), which reflect their changed constituents due to pronounced anthropogenic activity inputs. The result revealed that the Hg in the MR of the SNWDPC did not cause Hg pollution, however, the monitoring and control of risk sources around some stations with abnormally high measured concentrations should be enhanced.

The seasonal concentrations of Pb ranged from 0.2469 μg/L (winter) to 0.6338 μg/L (summer), while the annual mean concentration was 0.4304 μg/L. Since the sources of Pb were easily affected by human activities, the industrial production and recreational activities were more frequent in summer than in winter, which can lead to higher contents of Pb in the MR of the SNWDPC. Sixteen stations from the starting point TC to ZN had certain fluctuations of Pb, and these stations presented higher concentrations than the other 13 northern stations, from NC to HN (Figure 2b). The maximum detected Pb was at the LN and CQ stations (summer, 2.66 μg/L), while the highest annual mean concentration was 1.128 μg/L at the starting point TC station. Some studies have found that the Pb content in the Danjiangkou Reservoir was higher than that of other heavy metal ions that may lead to a higher annual mean concentration of Pb at the TC station than at the others in this study [45]. Considering that the LN and CQ stations are not geographically adjacent and are 177.8 kilometers apart (Figure 1), and that the Pb concentrations of these two stations in other seasons were quite low, the maximum Pb in summer primarily comes from external inputs. Considering the CQ station was downstream of the ZW station with a population of 9.88 million, the maximum detected Pb concentration at the CQ station reflected some understandably anthropogenic activity inputs between these two stations.

The annual mean concentration of As was 0.86 μg/L, and the seasonal concentrations ranged from 0.744 μg/L (autumn) to 1.10 μg/L (spring). The maximum detected (spring, 3.033 μg/L) and annual mean concentrations (1.567 μg/L) of As were both at the XF station. The distribution of As displayed a spatial trend that decreased from the southern to northern stations across the four seasons. From the starting point station TC to ZN, these 16 stations showed a certain volatility of As, while the other 13 stations, from NC to HN, showed significantly lower As contents than those of the southern stations (Figure 2c). Seasonal concentrations of Cd and Se were in the range of 0.0283 μg/L (winter) to 0.0303 μg/L (summer), and 0.299 μg/L (winter) to 0.310 μg/L (spring), respectively. The annual mean concentrations of Cd and Se were 0.0297 μg/L and 0.3025 μg/L, respectively. The maximum measured concentration of Cd was at the HW station (spring, 0.0833 μg/L), while the maximum concentration of Se was at the HN station (summer, 0.57 μg/L). The spatial variation trend of Cd was similar to As and Pb, with 16 stations from the starting point TC to ZN having higher concentrations than the 13 northern stations from the NC to HN (Figure 2d). There was no obvious spatial variation rule of Se in autumn and winter, however, in spring and summer, the Se of 13 stations displayed a fluctuating upward trend from NC to HN (Figure 2e).

The concentrations of Cr at all stations were very stable with no spatio-temporal variation across the four seasons (Figure 2f). Many real concentrations of Cr were actually undetected due to the low concentrations. According to the above-mentioned data processing rule, the annual mean concentration of Cr was 4.01 μg/L, which was lower than its Class I threshold (10 μg/L). Although these toxic metals are harmful to human health once exceeding certain standards [24], it can be seen that each metal was way below the threshold of Class I of their respective standard based on our analysis and showed relatively high levels of water quality in some international rivers. The results proved that there was no toxic-metals pollution in this project, but administrative departments should strengthen the control of risk sources at stations with abnormally high concentrations of some indicators.

#### 3.1.2. Easily Treated Indicators

**Figure 3:**
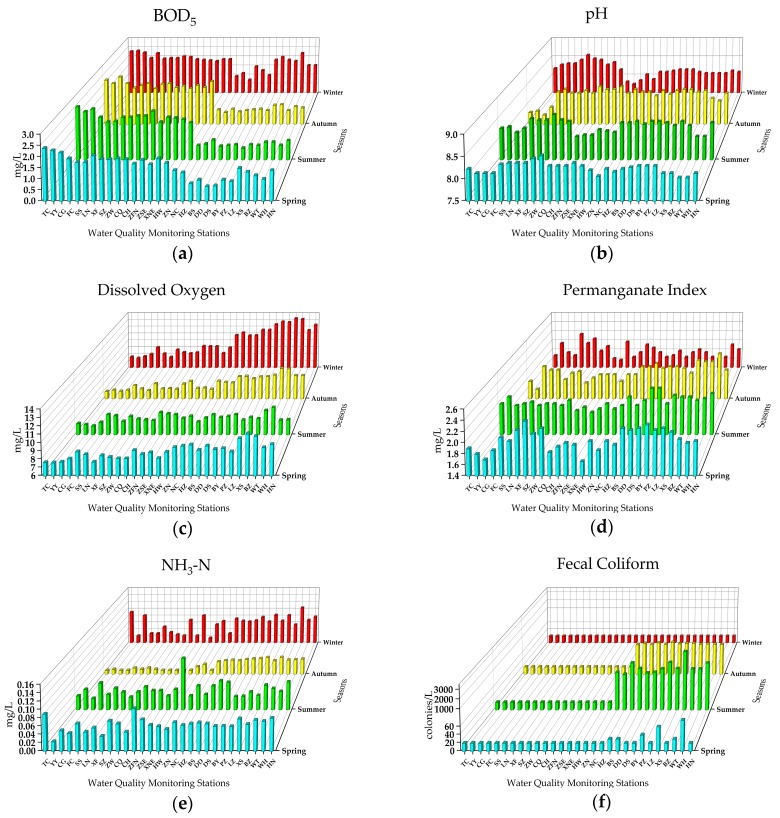
The concentrations of six easily treated indicators of the 29 stations in the MR of the SNWDPC.

The concentrations of six easily treated indicators are shown in Figure 3. In this group, five indicators, including pH, dissolved oxygen, permanganate index, NH_3_^–^ N, and fecal coliform, exhibited significant differences across the four seasons (one-way ANOVA, *P* < 0.05), while the BOD_5_ did not (one-way ANOVA, *P* > 0.05). The annual mean concentration of BOD_5_ was 1.56 mg/L, and the seasonal concentrations ranged from 1.47 mg/L (autumn) to 1.72 mg/L (winter). The maximum concentrations of the detected and annual mean were seen at TC station, with concentrations of 2.57 mg/L (summer) and 2.375 mg/L, respectively. The concentrations of BOD_5_ displayed a spatial distribution where the 16 stations from the starting point TC to the ZN had higher concentrations than the northern 13 stations (from NC to HN) across the four seasons (Figure 3a). Since the BOD_5_ can be used as an effective indicator to characterize the content of organic matter in water [52], the spatial distribution of BOD_5_ showed that the organic materials in this project could be reduced by self-purification after a high-flow water delivery through a long-distance open channel. The seasonal concentrations of pH ranged from 8.12 (winter) to 8.32 (summer), indicating that the water in the MR of the SNWDPC was weakly alkaline, and within the guideline of 6.0–9.0 recommended by the standard (No. GB3838-2002). The maximum detected pH was at the SZ station (summer, 8.6). The pH did not display obvious spatial variation from the southern to northern stations across the four seasons (Figure 3b). However, four consecutive stations (from TC to FC) had a pH lower than 8 in autumn, while there were five consecutive stations (from ZFN to ZN) in winter.

The annual average concentration of dissolved oxygen was 8.8 mg/L, which was in line with Class I of the standard. The lowest and highest seasonal concentrations of DO were in summer and winter, with concentrations of 8.19 mg/L and 9.88 mg/L, respectively. The maximum annual average concentration (10.92 mg/L) and detected concentrations (winter, 13.17 mg/L) of DO were both seen at the BZ station. DO presented an obvious fluctuating upward trend from the southern to northern stations across four seasons (Figure 3c). The dissolved oxygen of all stations in spring met the Class I standard, however, in autumn, winter, and summer, 11, one, and three stations detected DO concentrations at Class II, respectively. Three consecutive stations (from TC to CG) had an annual average content of DO at Class II, with concentrations <7.5 mg/L. The high concentrations of DO and the spatial variation of this indicator in the canal can be explained by two reasons: one is that the water quality of the water source was excellent and the content of oxygen-consuming organic matter in the water body was quite low, and the other one is that the high-flow water delivery through a long-distance open channel was in full contact with the air and the reoxygenation speed can be faster than in other natural water bodies, such as lakes and rivers [53]. Dissolved oxygen was generally negatively correlated with water temperature, i.e., high temperature and abundant sunshine are likely to cause lower concentrations of dissolved oxygen in summer than in winter [54]. Since dissolved oxygen is an important indicator to maintain biological survival in water and to measure the classifications of water quality [55], the interactions between dissolved oxygen and other water quality factors in this project need further study. In addition, some stations that detected dissolved oxygen concentrations increased abnormally in some seasons, therefore, there is a need to focus on algae density monitoring.

The permanganate index (PI) is a comprehensive indicator that can present the degree of the organic pollution of surface water [56]. There was no obvious spatial variation of PI from the southern to northern stations (Figure 3d). The annual mean concentration of PI was 1.95 mg/L, and the seasonal concentrations ranged from 1.76 mg/L (winter) to 2.05 mg/L (spring). There were two seasonal PI concentrations (spring and summer) higher than the threshold of Class I (2.00 mg/L), while 19, 19, 15, and two stations from spring to winter had PI concentrations >2.00 mg/L respectively, indicating that the MR of SNWDPC has a potential risk of organic pollution. The annual mean concentration of NH_3_^–^ N was 0.0493 mg/L, ranging from 0.0251 mg/L (autumn) to 0.0636 mg/L (spring). The highest annual average concentration (0.071 mg/L) and detected concentration (summer, 0.133 mg/L) were both at the HW station, revealing that some exogenous sources of nitrogen input at this station. The NH_3_^–^ N content in autumn was significantly lower than in other seasons, and the NH_3_^–^ N had no obvious spatial distribution from the southern to northern stations in spring, summer, and winter (Figure 3e).

The annual mean content of fecal coliform was 198 colonies/L, which was very close to the threshold of Class I (200 colonies/L), indicating that the FC has a high risk of becoming Class II. The maximum annual average (833 colonies/L) and the detected contents (3117 colonies/L, summer) were both at the BZ Station, while the highest seasonal content was in summer (630 colonies/L) (Figure 3f). Fecal coliform is a water quality indicator that is closely related to human activities. Since 13 stations (from NC to HN) pass through the densely populated areas of the Hebei Province, Tianjin Municipality, and Beijing, these northern stations are easily affected by intensive human activities, which led to significantly higher contents than those of the southern stations [57]. In addition, the water temperature was also a key factor that could cause the increase in the FC quantity. A low temperature environment is not conducive to the reproduction of FC, and high temperature in summer was a major cause of the amount of FC being significantly higher than in other seasons.

#### 3.1.3. Other Indicators

**Figure 4:**
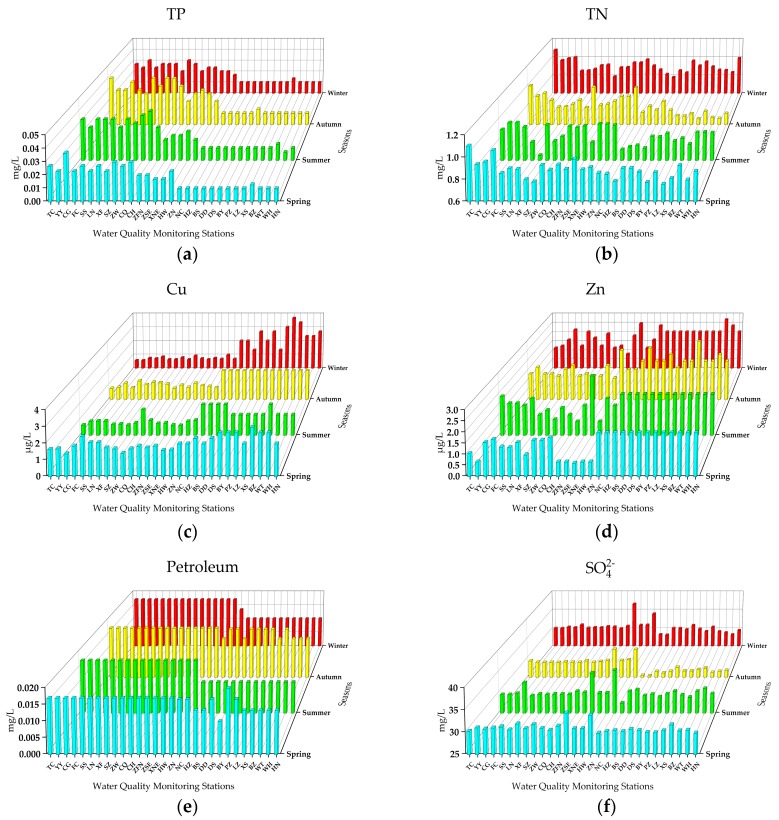

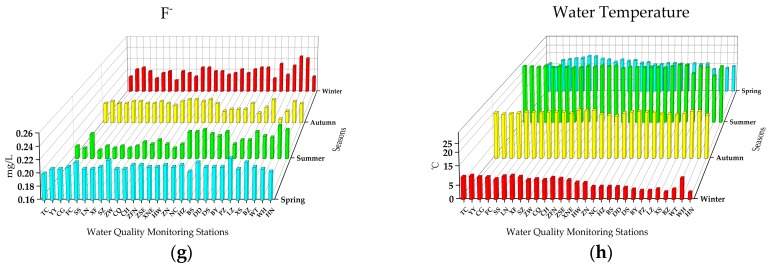
The concentrations of eight other indicators of the 29 stations in the MR of the SNWDPC.

Figure 4 presents the distributions and variations of eight indicators. Seven indicators including TN, TP, SO_4_^2−^, F^−^, Cu, petroleum, and water temperature had significant differences among the four seasons (one-way ANOVA, *P* < 0.05), while Zn did not (one-way ANOVA, *P* > 0.05).

The annual mean concentration of TP was 0.0195 mg/L, which was very close to the threshold of the Class I standard (0.02 mg/L), and the seasonal concentrations ranged from 0.0178 mg/L (winter) to 0.0220 mg/L (summer). There were two seasonal concentrations (summer and autumn) >0.02 mg/L, while 14, 14, 16, and 15 stations from spring to winter detected TP concentrations >0.02 mg/L, respectively. The TP concentrations showed a spatial trend that decreased from the southern to northern stations across the four seasons (Figure 4a). Starting from the TC station, 11 consecutive stations had annual mean concentrations >0.02 mg/L, while the maximum was at ZW station (0.034 mg/L). The maximum detected TP was at CQ station (summer and autumn, 0.04 mg/L). According to previous studies, the TP in the Danjiangkou Reservoir was maintained at the content level that was higher than 0.02 mg/L during the early operation period of the MR of the SNWDPC [58], which would cause the higher TP concentrations at the beginning of the canal. After long-distance delivery, the TP can be reduced due to the self-purification of the project by some physical, chemical, and biological processes in the water [59]. As about 80% of the eutrophication in water bodies is restricted by phosphorus [36], and nearly half of the stations in each season had TP concentrations exceeding the threshold of Class I, our results imply that besides the effect of the original water source, there were also some exogenous inputs that affect the TP concentrations, or can be closely related to strong human activities and the growth of algae [60]. Hence, the government should pay more attention to monitoring phosphorus sources and algae reproduction, especially in summer and autumn.

The annual mean concentration of TN was 0.86 mg/L, and the seasonal concentrations ranged from 0.804 mg/L (autumn) to 0.898 mg/L (winter). The maximum annual average (1.02 mg/L) and detected concentration (1.107 mg/L, spring) were both found at the TC station. The seasonal variation of TN and NH_3_^–^ N presented good similarity, with the concentrations gradually reduced from spring to autumn, and the lowest seasonal concentrations were both in autumn, while they increased significantly in winter (Figure 4b). The reason for this result can be attributed to the fact that algae proliferate more easily in summer and autumn under sufficient sunshine and high temperature, and absorb nitrogen in the water as a nutrient source, thus reducing the contents of TN and NH_3_^–^ N [61]. In winter, when the algae died and underwent microbial decomposition, large amounts of nitrogen was released and led to the increase of TN and NH_3_^–^ N. Related studies have shown that nitrogen and phosphorus are the key limiting factors for algal population density and quantity, but the dominant species of phytoplankton in different water environments have discrepancies, which would cause differences in nitrogen and phosphorus consumption [62]. The concentration ratio of TN to TP was 44.13:1, which indicates that the phosphorus was the limiting element of the density and quantity of algae in the MR of the SNWDPC, and the Danjiangkou Reservoir has a high risk of eutrophication due to the rich nitrogen and phosphorus [63].

The annual mean concentrations of Cu and Zn were 1.537 and 1.692 μg/L respectively, while the seasonal concentrations ranged from 1.185 μg/L (summer) to 2.053 μg/L (spring), and 1.539 μg/L (spring) to 1.786 μg/L (autumn), respectively. The maximum detected concentrations of Cu and Zn were both present at the XS station in autumn, with concentrations of 3.667 and 3.010 μg/L, respectively. Cu exhibited spatial variation where the concentrations of 13 northern stations from NC to HN showed a general upward trend that was higher than those of the 16 southern stations from TC to ZN (Figure 4c). The Zn content in the 13 northern stations from NC to HN was relatively stable with no significant seasonal fluctuations, while 16 southern stations from the TC to ZN showed obvious fluctuation across the four seasons (Figure 4d). As the contents of Cu and Zn were far lower than the respective threshold concentration of Class I, and there was no abnormally high concentration point, this has proven that the MR of the SNWDPC has no risk of Cu and Zn pollution.

The concentration of petroleum at all stations was very stable, most of the petroleum concentrations were actually undetected and had no spatio-temporal variations in the four seasons (Figure 4e). The SO_4_^2−^ and F^−^ fluctuated from the southern to northern stations in the four seasons, and their spatial distributions had no obvious regularity (Figure 4f,g). The annual mean concentrations of SO_4_^2−^ and F^−^ were 29.86 and 0.197 mg/L respectively, while the seasonal concentrations ranged from 28.16 mg/L (autumn) to 30.98 mg/L (spring), and 0.190 mg/L (summer) to 0.210 mg/L (spring), respectively. Since SO_4_^2−^ and F^−^ are important soluble inorganic salts that can reflect the load of inorganic nutrient pollutants in water to some extent [64], stations with abnormally high concentrations such as ZSE and ZN should focus on monitoring and researching the sources of inorganic nutrients.

The water temperature gradually decreased from the southern to northern stations, which is consistent with the geographic variation of air temperature in China (Figure 4h). The annual average of water temperature is 17.55 °C, and the seasonal concentrations range from 6.12 °C (winter) to 27.78 °C (summer). Water temperature can affect the contents of DO, BOD_5_, and the vertical distribution of many inorganic salts [65], and can directly change the nitrogen and phosphorus cycle processes [6,62,63]. The relationships and interactions of water temperature and other water quality indicators need further study.

### 3.2. Statistical Analyses

#### 3.2.1. Correlation Analysis

**Figure 5:**
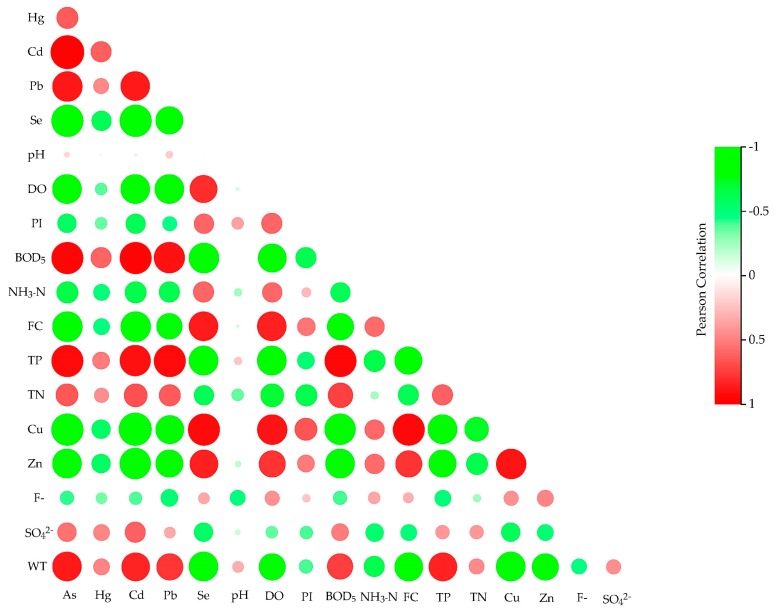
Pearson correlation coefficients of the water quality indicators in the MR of the SNWDPC.

The Pearson correlation was used to study and understand the relationships and interactions between the water quality indicators in the MR of the SNWDPC. Since the concentrations of Cr and Petroleum were stable and undetected at most stations, their correlations were not calculated and analyzed in this study. The impacts of different hydrological, geochemical, and human activities on the water quality at the different stations could be responsible for strong or weak correlation coefficients. The results are shown in Table 5 and Figure 5.

Table 5 and Figure 5 display the correlation coefficients between each pair of indicators at the 0.01 or 0.05 probability level. There were significant relationships between the five metal indicators, i.e., the As, Pb, Hg, and Cd had positive correlation coefficients of 0.465 to 0.977 at the 0.01 level, and Se had negative correlation coefficients with those four indicators, with values ranging from −0.598 to −0.934 at the 0.01 level, indicating that the contents of As, Pb, Hg, and Cd in the MR of the SNWDPC are mainly from sources affected by human activities, which is basically consistent with previous studies [5,13,24], while the inverse relationship of Se with As, Pb, Hg, and Cd was indicative of the exogenous inputs of natural sources [12]. As there were no statistically significant correlations between pH and other indicators except PI and F^−^, which could be due to the fact that the pH was maintained in a range of 8.12 to 8.32 throughout the monitoring year, this indicates that the physical, chemical, and biological reactions and the growth process of aquatic organisms in this project had a stable alkaline environment [66], thus the pH did not become a limiting indicator in this study [67]. Since the effects of pH on other indicators cannot be determined by the correlation coefficients, the interactions between pH and planktonic algae or other water quality indicators in the project need further study. The BOD_5_ indicates the degree of organic pollution in water body, i.e., the higher the BOD_5_ concentrations, the more dissolved oxygen is consumed by microbial metabolism, hence the BOD_5_ generally has a strong a negative correlation with dissolved oxygen (−0.829, *P* < 0.01) [52]. High temperature and sunshine duration in summer easily lead to the escape of dissolved oxygen in water [65], so the DO generally has a negative correlation with water temperature (−0.798, *P* < 0.01). Moreover, when the dissolved oxygen content was high, the sediment in water was at an oxidation state that would be suitable for the growth of aerobic bacteria such as nitrobacteria and nitrifying bacteria, and can accelerate the nitrogen and phosphorous cycle processes [62], resulting in the DO having a negative correlation with TN (−0.688, *P* < 0.01) and TP (−0.870, *P* < 0.01). In addition, higher dissolved oxygen contents also present a stronger self-purification ability of water, which would accelerate the oxidation reaction of metal ions and make them precipitate with sediment [17], so the As, Pb, Hg, and Cd will always have negative and positive correlation coefficients with dissolved oxygen and BOD_5_ respectively, and a sufficient dissolved oxygen environment will also lead to the increase of fecal coliform [57]. These relationships, as reflected in the correlation coefficients, were basically consistent with previous research and natural phenomena.

The correlation coefficients of TP and TN with other indicators presented good similarity, but there were more significant statistical relationships between the TP and other indicators. This result also indicates that the TP was the key limiting indicator to characterize the nutritional status of water quality in the MR of the SNWDPC, which is consistent with our previous analysis [63,68]. The correlation coefficients of Cu and Zn with other indicators were basically consistent with Se, which suggests that these indicators predominantly originate from similar sources. Although the SO_4_^2−^ and F^−^ are important soluble inorganic salts that can reflect the load of inorganic nutrient pollutants in water, they both had weak correlation coefficients with most of the indicators in this case, which are indictive of various factors and multiple sources [64].

#### 3.2.2. Principal Component Analysis

**Figure 6:**
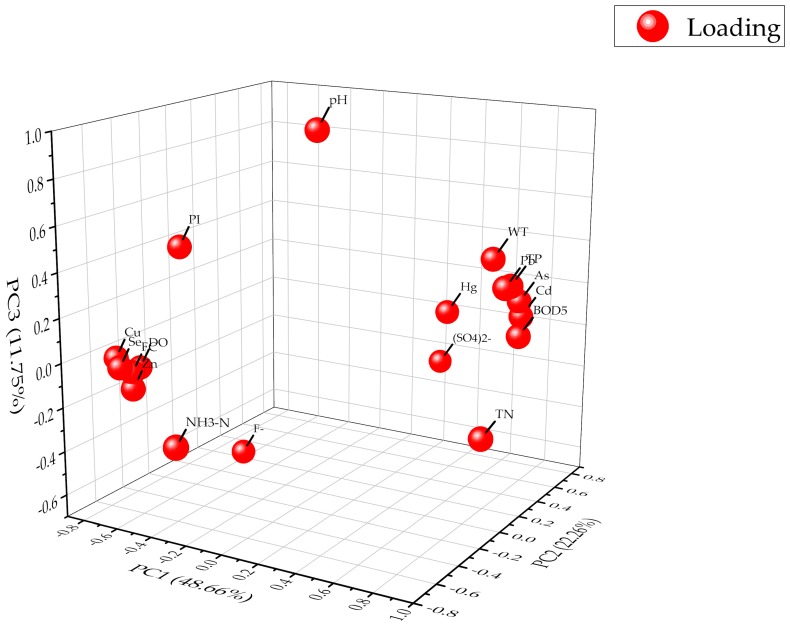
The principal component analysis (PCA) loadings of the water quality indicators in the MR of the SNWDPC.

In this study, the PCA method was applied to identify the most contributive natural and anthropogenic sources of the indicators, and to further understand their distribution characteristics. Data reliability for the PCA was calculated by the KMO (Kaiser–Meyer–Olkin) test and Bartlett’s test of sphericity. The results are presented in Table 6 in Figure 6.

The results showed that the KMO test value was 0.846 and the $\chi^{2}$ of Bartlett’s test was 676.95 at the significance level of *P* < 0.01, which proved the suitability of the data for PCA. Three components with eigenvalues >1 explained about 82.67% of the total variance (Table 6). Eighteen indicators were assembled into three groups. The first principal component (PC) accounting for the most variance (48.66%) had high loadings with absolute values from 0.674 to 0.855 on 13 indicators, including As, Cd, Pb, Se, DO, PI, BOD_5_, FC, TP, TN, Cu, Zn, and WT, presented with a very close distance of the loading balls in Figure 6. The PI, DO, BOD_5_, FC, TP, and TN mainly reflected the effects of the organic pollution, nutrients, and bacteria caused by human activities, i.e., PI, DO, and BOD_5_ are indicators to measure the pollution degree of the surface water by organic and reductive inorganic substances [52]. Fecal coliform has been widely used to indicate the extent of fecal contamination and has been reported to be strongly positively correlated with pathogenic intestinal bacteria [39], and TN and TP are the most important indicators to reflect the human activities elevating nutrient levels in water bodies [60,61]. As, Cd, Pb, Cu, and Zn are primarily contributable to human activities, such as vehicle exhaust emissions [14], mining engineering [9], and metal production [13], while the content of Se can be influenced by mineral or crustal weathering [16] and pedogenesis [17], hence the first PC was indicative of the mixed sources of anthropogenic and natural contributions.

The second and third PCs explained 22.26% and 11.75% of the total variance respectively, and were mainly contributed by Hg, NH_3_^–^ N, SO_4_^2−^, and pH and F- respectively, which corresponded to the relatively high loadings on Axes 2 and 3, respectively (Figure 6). The Hg predominantly came from agrochemicals and industrial waste [10,11,69], while NH_3_^–^ N and SO_4_^2−^ are primarily derived from some soluble inorganic nitrogen and inorganic salts affected by some agricultural practices and mining engineering [6,12]. Therefore, the second PC was assumed to come from mixed exogenous sources of human activities. However, the inverse loading relationships of Hg and SO_4_^2−^ with NH_3_^–^ N were suggestive of the external inputs of Hg and SO_4_^2−^. The third PC had high loadings on pH (0.939) and F^−^ (−0.608), which reflects the physical and soluble inorganic salt characteristics of the water quality. Since the F^−^ was principally from some natural processes, such as mineral weathering and karstification, this component was ascribed to natural sources [64].

The PCA results suggest that the impacts of natural and human activities on the water quality in the MR of the SNWDPC are relatively complex. Although the project has adopted various strict protections to prevent the external sources of pollution, the impact of anthropogenic activities, especially those of industrial and agricultural activities on water quality, cannot be ignored. Therefore, the government should conduct further investigation and control measurements of risk sources in intense anthropogenic areas.

### 3.3. Water Quality Assessment

**Figure 7:**
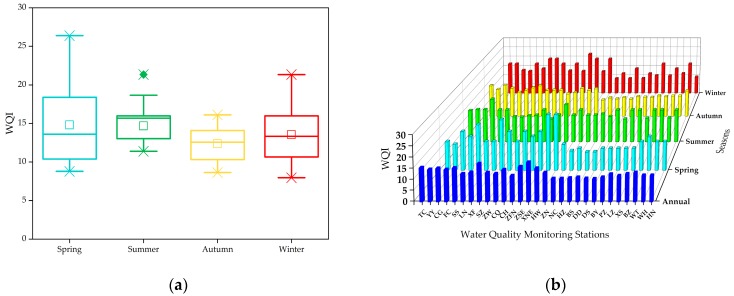
The WQI values of the 29 water quality monitoring stations in the MR of the SNWDPC. (**a**) The box plot of the seasonal and annual mean values of WQI; (**b**) The seasonal variation of the WQI.

The WQI calculation results are presented in Figure 7. The annual mean value of WQI was 13.24, and the values of the 29 stations ranged from 10.49 to 17.93, while the seasonal concentrations from spring to winter were 14.80, 14.68, 12.36, and 13.54 (Figure 7a) respectively, which revealed that the water quality in the MR of the SNWDPC was in line with the “Excellent” level (Table 3) and was quite safe and suitable as a source of water and in national protection areas.

However, the maximum calculated WQIs across the four seasons were 26.40, 21.34, 16.13, and 21.33 respectively, and six stations had water quality at the “Good” level, with WQI values > 20 including four (SS, SZ, XNE, and HW stations) in spring, one (FC station) in summer, and one (ZSE station) in winter (Figure 7a,b), while spring had the largest WQI change interval, with a value of 8.79 to 26.40. These results indicate that the water quality have higher risk in spring than other seasons. The calculation results also showed that the WQIs of these stations were all determined by the Hg indicator due to its corresponding highest $I_{i}$ value in the Toxic Metals Group. Although the maximum detected Hg value in 2016 was 0.033 μg/L, which was still lower than the threshold of Class I, however, when the content of Hg exceeded 0.05 μg/L, the water would directly become Class III (Table 2). More importantly, almost all forms of Hg in water can be converted to methylmercury with appropriate conditions of temperature, pH, and dissolved oxygen, which would easily transfer and accumulate in the food chain of aquatic systems and can threaten human health, such as damage to the human nervous system and harm the fetus, therefore, the monitoring should pay special attention to the Hg [39]. Our results also suggest that the management departments should strengthen the investigation and supervision to control the urban and industrial production and risk sources within the areas of high-density human activities along the MR of the SNWDPC, and carry out corresponding treatments to reduce the impacts of potential pollutants carried by rainfall runoff and dust fall on water quality.

## 4. Conclusions

This article presented 6960 observations of 20 water quality indicators in 29 water quality monitoring stations of the South-to-North Water Diversion Project of China in 2016 to analyze the spatio-temporal characterization of water quality indicators, identify the main contributive anthropogenic and natural sources impact on the indicators, and make full use of the data to evaluate and understand the water quality classifications and their variation. Our conclusions can be presented as follows:

1. Six toxic metals including Hg, Pb, As, Cd, Se, and Cr of the 29 water quality monitoring stations were shown to have quite low concentrations that were all in line with the Class I standard. The As, Pb, and Cd presented spatial variations in 16 southern stations (from TC to ZN) which had higher concentrations than the rest of the northern stations, revealing that more detection should be put into these southern stations. Biochemical indicators including the dissolved oxygen, BOD_5_, permanganate index, ammonia nitrogen, and fecal coliform met the Class I or Class II levels, while the dissolved oxygen and BOD_5_ showed opposite spatial variability in the Easily Treated Indicators group. The concentration ratio of the TN to TP was 44.13:1 and these two indicators had relatively higher concentrations than the other nutrient factors, which indicated that the project has a potential risk of eutrophication and corresponding treatments should be considered.

2. The one-way analysis of variance results demonstrated that there were 14 indicators including the Hg, Pb, pH, dissolved oxygen, permanganate index, NH_3_^–^ N, fecal coliform, TN, TP, SO_4_^2−^, F^−^, Cu, petroleum, and water temperature exhibited significant differences across the four seasons. Four toxic metals indicators, including As, Pb, Hg, and Cd, had positive correlation coefficients ranging from 0.465 to 0.977 between each pair at the *P* < 0.01 level, while the TP and TN presented good similarity with other indicators according to the correlation analysis results.

3. The calculations of PCA showed that the first principal component with 48.66% of the total variance was controlled by mixed sources of anthropogenic and natural contributions, while the second principal component had high loadings on Hg, NH_3_^–^ N, and SO_4_^2−^, showing that 22.26% of the total variance mainly came from anthropogenic inputs, and the F- was mainly impacted by natural sources.

4. The WQI result revealed that the average WQI value of this project was 13.24, which corresponded to an “Excellent” level of water quality. There were six stations in total with WQI values exceeding 20 across different seasons, with a rating of “Good” in terms of water quality. Since the WQI values that exceeded 20 were all determined by the contents of Hg in this study, the result also indicates that the potential risk of industrial and agricultural activities near corresponding stations with abnormal WQI values should be carefully investigated, especially sources of Hg, and the monitoring of water quality should be strengthened.

5. The dominant species and the density of algae and their relationship with the water quality indicators of the MR of the SNWDPC are required for further research, and the interactions of water velocity and flow with the water quality indicators are also needed for greater quantificational study in the future for more efficient water quality management.

## Figures and Tables

**Table 1:** The locations of 29 fixed water quality monitoring stations of the Middle Route (MR) of the South-to-North Water Diversion Project of China (SNWDPC).

Province/Municipality	City/County	Population (×10^4^)	Stations	Code
Henan	Xichuan County	68.56	Taocha	TC
	Dengzhou City	178.6	Yaoying	YY
	Nanyang City	863.4	Chenggou	CG
	Fangcheng County	102.8	Fangcheng	FC
	Lushan County	78.16	Shahe South	SS
	Shan County	35.01	Lanhe North	LN
	Yuzhou City	114.87	Xinfeng	XF
	Xizheng County	63.58	Suzhang	SZ
	Zhengzhou City	988.1	Zhengwan	ZW
	Xingyang County	61.58	Chuanhuangqian	CQ
	Zhengzhou City	988.1	Chuanhuanghou	CH
	Hui County	75.13	Zhifanghe North	ZFN
	Hui County	75.13	Zhaozhuang Southeast	ZSE
	Weihui County	48.59	Xisimen Northeast	XNE
	Anyang City	512.85	Houxiaotun West	HW
	Anyang County	85.42	Zhanghe North	ZN
Hebei	Ci County	65	Nanyingcun	NC
	Shahe City	44.52	Houzhuang	HZ
	Lincheng County	20.41	Beipanshi	BS
	Lincheng County	20.41	Dongdu	DD
	Shijiazhuang City	1,078.46	Daanshe	DS
	Xinle City	48.8	Beidayue	BY
	Shunping County	32.1	Puwangzhuang	PZ
	Mancheng County	42.2	Liujiazuo	LZ
	Xushui County	61.01	Xiheishan	XS
	Bazhou City	56.62	Bazhou	BZ
Tianjin Municipality	Wuqing District	119.96	Wangqingtuo	WT
	Xiqing District	85.37	Waihuanhe	WH
Beijing Capital	Fangshan District	109.6	Huinanzhuang	HN

**Table 2:** The classification and assessment standards of water quality indicators.

Classification	Weight (*P_i_*)	Indicators	Surface Water Environmental Quality Standards				
			I	II	III	IV	V
			$I_{i,1}\;$= 20	$I_{i,2}\;$= 40	$I_{i,3\;}$= 60	$I_{i,4}\;$= 80	$I_{i,5}\;$= 100
Toxic metals	-	As/μg L^−1^ ≤	50	50	50	100	100
	-	Hg/μg L^−1^ ≤	0.05	0.05	0.1	1	1
	-	Cd/μg L^−1^ ≤	1	5	5	5	10
	-	Cr/μg L^−1^ ≤	10	50	50	50	100
	-	Pb/μg L^−1^ ≤	10	10	50	50	100
	-	Se/μg L^−1^ ≤	10	10	10	20	20
Easily treated parameters	1	pH	6~9				
	4	DO/mg L^−1^ ≥	7.5	6	5	3	2
	3	PI/mg L^−1^ ≤	2	4	6	10	15
	3	BOD_5_/mg L^−1^ ≤	3	3	4	6	10
	3	NH_3_^–^ N /mg L^−1^ ≤	0.15	0.5	1	1.5	2
	3	FC/colonies L^−1^ ≤	200	2,000	10,000	20,000	40,000
Other parameters	4	TP/mg L^−1^ ≤	0.02	0.1	0.2	0.3	0.4
	-	TN*/mg L^−1^ ≤	0.2	0.5	1	1.5	2
	2	Cu/μg L^−1^ ≤	10	1000	1000	1000	1000
	2	Zn/μg L^−1^ ≤	50	1000	1000	2000	2000
	2	F^-^/mg L^−1^ ≤	1	1	1	1.5	1.5
	2	Petroleum/ mg L^−1^ ≤	0.05	0.05	0.05	0.5	1
	-	WT/°C	Maximum weekly average rise ≤ 1, drop ≤ 2.				
	2	SO_4_^2−^/mg L^−1^ ≤	250				

* The classification standard of TN is only applicable to the evaluation of lakes and reservoirs, so the water quality index (WQI) calculation in this study did not include the TN.

**Table 3:** The water quality classification according to WQI values.

WQI Value	≤20	21–40	41–60	61–80	81–100
Water Quality	Excellent	Good	Medium	Poor	Very Poor

**Table 4:** A comparison of the concentrations of toxic metal indicators in the MR of the SNWDPC with other studies and guidelines.

	As	Hg	Pb	Cd	Se	Cr
Max	3.033	0.033	2.660	0.083	0.567	2.667
Min	0.133	0.007	nd	nd	0.200	5.333
Mean	0.860	0.016	0.430	0.030	0.302	4.009
SD	0.612	0.006	0.593	0.028	0.083	0.189
Danjiangkou Reservoir, China [46]	0.86	nd	0.76	0.015	0.13	0.26
Han Jiang River, China [12]	14.42	nd	nd	2.31	nd	8.14
Three Gorges Reservoir, China [47]	nd	0.018	3.244	1.125	nd	10.12
Background, Dongting lake, China [48]	0.9	0.025	1	0.06	nd	0.89
Dil Deresi, Turkey [49]	50	nd	120	8	nd	42
WHO ^a^	10	1	10	3	10	50
US EPA MCL ^b^	10	2	15	5	50	100

^a^ World Health Organization Drinking Water Guidelines (2017) [50]. ^b^ United States Environmental Protection Agency [51].

**Table 5:** Pearson correlation matrix of the water quality indicators of the 29 stations in the MR of the SNWDPC.

	As	Hg	Cd	Pb	Se	pH	DO	PI	BOD_5_	NH_3_^–^ N	FC	TP	TN	Cu	Zn	F-	SO_4_^2−^	WT
**As**	1																	
**Hg**	**0.639** ^a^	1																
**Cd**	**0.977** ^a^	**0.624** ^a^	1															
**Pb**	**0.881** ^a^	**0.465** ^a^	**0.875** ^a^	1														
**Se**	**−0.930** ^a^	**−0.598** ^a^	**−0.934** ^a^	**−0.818** ^a^	1													
**pH**	0.165	0.056	0.100	0.208	−0.033	1												
**DO**	**−0.860** ^a^	**−0.373** ^b^	**−0.858** ^a^	**−0.869** ^a^	**0.812** ^a^	−0.120	1											
**PI**	**−0.566** ^a^	−0.354	**−0.593** ^a^	**−0.446b**	**0.598** ^a^	**0.369** ^a^	**0.602** ^a^	1										
**BOD_5_**	**0.942** ^a^	**0.606** ^a^	**0.946** ^a^	**0.901** ^a^	**−0.881** ^a^	−0.006	**−0.829** ^a^	**−0.613** ^a^	1									
**NH_3_–N**	**−0.644** ^a^	**−0.498** ^a^	**−0.635** ^a^	**−0.619** ^a^	**0.603** ^a^	−0.249	**0.591** ^a^	0.275	**−0.599** ^a^	1								
**FC**	**−0.875** ^a^	**−0.479** ^a^	**−0.880** ^a^	**−0.771** ^a^	**0.874** ^a^	−0.115	**0.858** ^a^	**0.542** ^a^	**−0.794** ^a^	**0.578** ^a^	1							
**TP**	**0.929** ^a^	**0.524** ^a^	**0.904** ^a^	**0.928** ^a^	**−0.863** ^a^	0.228	**−0.870** ^a^	**−0.503** ^a^	**0.936** ^a^	**−0.633** ^a^	**−0.812** ^a^	1						
**TN**	**0.655** ^a^	**0.445** ^b^	**0.683** ^a^	**0.641** ^a^	**−0.596** ^a^	−0.362	**−0.688** ^a^	**−0.626** ^a^	**0.741** ^a^	−0.239	**−0.609** ^a^	**0.623** ^a^	1					
**Cu**	**−0.942** ^a^	**−0.561** ^a^	**−0.954** ^a^	**−0.840** ^a^	**0.927** ^a^	−0.047	**0.893** ^a^	**0.666** ^a^	**−0.902** ^a^	**0.588** ^a^	**0.930** ^a^	**−0.869** ^a^	**−0.724** ^a^	**1**				
**Zn**	**−0.875** ^a^	**−0.570** ^a^	**−0.917** ^a^	**−0.807** ^a^	**0.848** ^a^	−0.180	**0.789** ^a^	**0.521** ^a^	**−0.859** ^a^	**0.577** ^a^	**0.788** ^a^	**−0.819** ^a^	**−0.630** ^a^	**0.896** ^a^	**1**			
**F-**	**−0.418** ^b^	−0.331	**−0.388** ^b^	**−0.509** ^a^	0.342	**−0.472** ^a^	**0.433** ^b^	0.234	**−0.406** ^b^	0.351	0.319	**−0.491** ^a^	−0.243	**0.434** ^b^	**0.479** ^a^	1		
**SO_4_^2^**	**0.558** ^a^	**0.478** ^a^	**0.617** ^a^	0.339	**−0.556** ^a^	−0.152	−0.363	**−0.397** ^b^	**0.511** ^a^	**−0.525** ^a^	**−0.490** ^a^	**0.405** ^a^	**0.406** ^b^	**−0.575** ^a^	**−0.499** ^a^	0.01	1	
**WT**	**0.875** ^a^	**0.486** ^a^	**0.833** ^a^	**0.777** ^a^	**−0.859** ^a^	0.328	**−0.798** ^a^	**−0.402** ^b^	**0.751** ^a^	**−0.621** ^a^	**−0.828** ^a^	**0.841** ^a^	**0.460** ^b^	**−0.855** ^a^	**−0.780** ^a^	**−0.457** ^b^	**0.442** ^a^	1

Bold values represent correlation with significance. ^a^ Significance at the 0.01 probability level. ^b^ Significance at the 0.05 probability level.

**Table 6:** The component matrix of the water quality indicators in the MR of the SNWDPC.

**Component**	**Initial Eigen Values**			**Rotation Sums of Squared Loadings**		
	**Total**	**% of variance**	**Cumulative %**	**Total**	**% of variance**	**Cumulative %**
1	11.872	65.958	65.958	8.759	48.664	48.664
2	1.925	10.697	76.655	4.006	22.258	70.922
3	1.083	6.018	82.673	2.115	11.752	82.673
4	0.768	4.268	86.941			
5	0.521	2.895	89.836			
**Variables**	**Component**					**Communalities**
	**1**		**2**	**3**		
As	**0.793**		0.550	0.167		0.959
Hg	0.347		**0.639**	0.040		0.530
Cd	**0.796**		0.568	0.094		0.966
Pb	**0.832**		0.313	0.289		0.873
Se	**−0.763**		−0.553	−0.066		0.892
pH	−0.077		0.094	**0.939**		0.897
DO	**−0.880**		−0.267	−0.160		0.871
PI	**−0.720**		−0.142	0.395		0.695
BOD_5_	**0.845**		0.446	0.039		0.915
NH_3_^–^ N	−0.318		**−0.706**	−0.312		0.698
FC	**−0.761**		−0.466	−0.112		0.809
TP	**0.822**		0.394	0.278		0.908
TN	**0.819**		0.131	−0.348		0.809
Cu	**−0.855**		−0.471	−0.057		0.956
Zn	**−0.764**		−0.461	−0.190		0.832
F-	−0.501		0.061	**−0.608**		0.625
SO_4_^2−^	0.202		**0.839**	−0.275		0.820
WT	**0.674**		0.491	0.362		0.827

Bold absolute values are > 0.6 [25].

## References

[1] Vorosmarty C.J., McIntyre P.B., Gessner M.O., Dudgeon D., Prusevich A., Green P., Glidden S., Bunn S.E., Sullivan C.A., Liermann C.R. (2010). Global threats to human water security and river biodiversity. Nature.

[2] Tiwari A.K., Singh A.K., Mahato M.K. (2018). Assessment of groundwater quality of pratapgarh district in india for suitability of drinking purpose using water quality index (wqi) and gis technique. Sustain. Water Resour. Manag..

[3] Liang B., Han G.L., Liu M., Yang K.H., Li X.Q., Liu J.K. (2018). Distribution, sources, and water quality assessment of dissolved heavy metals in the jiulongjiang river water, southeast China. Int. J. Environ. Res. Public Health.

[4] Zhang Y.C., Ma R.H., Hu M.Q., Luo J.H., Li J., Liang Q.C. (2017). Combining citizen science and land use data to identify drivers of eutrophication in the huangpu river system. Sci. Total Environ..

[5] Chowdhury S., Mazumder M.A.J., Al-Attas O., Husain T. (2016). Heavy metals in drinking water: Occurrences, implications, and future needs in developing countries. Sci. Total Environ..

[6] Hou W., Gu B.H., Lin Q.Q., Gu J.G., Han B.P. (2013). Stable isotope composition of suspended particulate organic matter in twenty reservoirs from guangdong, southern China: Implications for pelagic carbon and nitrogen cycling. Water Res..

[7] Todd A.S., Manning A.H., Verplanck P.L., Crouch C., McKnight D.M., Dunham R. (2012). Climate-change-driven deterioration of water quality in a mineralized watershed. Environ. Sci. Technol..

[8] Xiao J., Wang L.Q., Deng L., Jin Z.D. (2019). Characteristics, sources, water quality and health risk assessment of trace elements in river water and well water in the chinese loess plateau. Sci. Total Environ..

[9] Soleimani H., Nasri O., Ojaghi B., Pasalari H., Hosseini M., Hashemzadeh B., Kavosi A., Masoumi S., Radfard M., Adibzadeh A. (2018). Data on drinking water quality using water quality index (wqi) and assessment of groundwater quality for irrigation purposes in qorveh&dehgolan, kurdistan, iran. Data Brief.

[10] Abbasnia A., Radfard M., Mahvi A.H., Nabizadeh R., Yousefi M., Soleimani H., Alimohammadi M. (2018). Groundwater quality assessment for irrigation purposes based on irrigation water quality index and its zoning with gis in the villages of chabahar, sistan and baluchistan, iran. Data Brief.

[11] Wang X.L., Lu Y.L., Han J.Y., He G.Z., Wang T.Y. (2007). Identification of anthropogenic influences on water quality of rivers in taihu watershed. J. Environ. Sci..

[12] Li S.Y., Zhang Q.F. (2010). Spatial characterization of dissolved trace elements and heavy metals in the upper han river (China) using multivariate statistical techniques. J. Hazard. Mater..

[13] Pertsemli E., Voutsa D. (2007). Distribution of heavy metals in lakes doirani and kerkini, northern greece. J. Hazard. Mater..

[14] Neal C., Neal M., Hill L., Wickham H. (2006). The water quality of the river thame in the thames basin of south/south-eastern england. Sci. Total Environ..

[15] Mohebbi M.R., Saeedi R., Montazeri A., Vaghefi K.A., Labbafi S., Oktaie S., Abtahi M., Mohagheghian A. (2013). Assessment of water quality in groundwater resources of iran using a modified drinking water quality index (dwqi). Ecol. Indic..

[16] Solovieva N., Jones V.J., Appleby P.G., Kondratenok B.M. (2002). Extent, environmental impact and long-term trends in atmospheric contamination in the usa basin of east-european russian arctic. Water Air Soil Pollut..

[17] Cheung K.C., Poon B.H.T., Lan C.Y., Wong M.H. (2003). Assessment of metal and nutrient concentrations in river water and sediment collected from the cities in the pearl river delta, south China. Chemosphere.

[18] Putro B., Kjeldsen T.R., Hutchins M.G., Miller J. (2016). An empirical investigation of climate and land-use effects on water quantity and quality in two urbanising catchments in the southern united kingdom. Sci. Total Environ..

[19] Paerl H.W., Xu H., McCarthy M.J., Zhu G.W., Qin B.Q., Li Y.P., Gardner W.S. (2011). Controlling harmful cyanobacterial blooms in a hyper-eutrophic lake (lake taihu, China): The need for a dual nutrient (n & p) management strategy. Water Res..

[20] Lumb A., Sharma T.C., Bibeault J.F. (2011). A review of genesis and evolution of water quality index (wqi) and some future directions. Water Qual. Expos. Health.

[21] Wang Z.M., Shao D.G., Yang H.D., Yang S. (2015). Prediction of water quality in south to north water transfer project of China based on ga-optimized general regression neural network. Water Sci. Technol.-Water Supply.

[22] Shao D.G., Wang Z.M., Wang B., Luo W.W. (2016). A water quality model with three dimensional variational data assimilation for contaminant transport. Water Resour. Manag..

[23] Zhao P., Tang X.Y., Tang J.L., Wang C. (2013). Assessing water quality of three gorges reservoir, China, over a five-year period from 2006 to 2011. Water Resour. Manag..

[24] Li J.L., He M., Han W., Gu Y.F. (2009). Analysis and assessment on heavy metal sources in the coastal soils developed from alluvial deposits using multivariate statistical methods. J. Hazard. Mater..

[25] Kocer M.A.T., Sevgili H. (2014). Parameters selection for water quality index in the assessment of the environmental impacts of land-based trout farms. Ecol. Indic..

[26] Fan C., Wang G.S., Chen Y.C., Ko C.H. (2009). Risk assessment of exposure to volatile organic compounds in groundwater in taiwan. Sci. Total Environ..

[27] Sun W., Xia C.Y., Xu M.Y., Guo J., Sun G.P. (2016). Application of modified water quality indices as indicators to assess the spatial and temporal trends of water quality in the dongjiang river. Ecol. Indic..

[28] Rubio-Arias H., Contreras-Caraveo M., Manuel Quintana R., Alfonso Saucedo-Teran R., Pinales-Munguia A. (2012). An overall water quality index (wqi) for a man-made aquatic reservoir in mexico. Int. J. Environ. Res. Public Health.

[29] da Silva G.S., Jardim W.D.F. (2006). A new water quality index for protection of aquatic life appllied to the atibaia river, region of campinas/paulinia cities—Sao paulo state. Quim. Nova.

[30] Deng K., Wang J.G., Wang J.Q., Wang C.X., Zhang C.J., Wang X.Y. (2015). Treatments to control urban river pollution in water source city of south to north water diversion project. Pol. J. Environ. Stud..

[31] Zhang P., Tian B. (2011). Real time temperature simulation on caohe river aqueduct in south to north water transfer project during construction period. Adv. Civ. Eng..

[32] Zhang Z.W., Zeng H., Guo J. The mechanism of environmental protection of water resource area of the middle route of chinese south to north water transfer project. Proceedings of the International Conference on Engineering And Business Management (Ebm2011).

[33] Li L.C., Zhang L.P., Xia J., Gippel C.J., Wang R.C., Zeng S.D. (2015). Implications of modelled climate and land cover changes on runoff in the middle route of the south to north water transfer project in China. Water Resour. Manag..

[34] Sener S., Sener E., Davraz A. (2017). Evaluation of water quality using water quality index (wqi) method and gis in aksu river (sw-turkey). Sci. Total Environ..

[35] BengraïNe K., Marhaba T.F. (2003). Using principal component analysis to monitor spatial and temporal changes in water quality. J. Hazard. Mater..

[36] Primpas I., Tsirtsis G., Karydis M., Kokkoris G.D. (2010). Principal component analysis: Development of a multivariate index for assessing eutrophication according to the european water framework directive. Ecol. Indic..

[37] Dutta S., Dwivedi A., Kumar M.S. (2018). Use of water quality index and multivariate statistical techniques for the assessment of spatial variations in water quality of a small river. Environ. Monit. Assess..

[38] Xiao K.H., Yang J., Li Y.X., Quan Q.M. (2019). Temporal and spatial variations in water quality of changjiang river basin in luzhou, China based on multivariate statistical techniques. Desalin. Water Treat..

[39] Hou W., Sun S.H., Wang M.Q., Li X., Zhang N., Xin X.D., Sun L., Li W., Jia R.B. (2016). Assessing water quality of five typical reservoirs in lower reaches of yellow river, China: Using a water quality index method. Ecol. Indic..

[40] Wu Z.S., Zhang D.W., Cai Y.J., Wang X.L., Zhang L., Chen Y.W. (2017). Water quality assessment based on the water quality index method in lake poyang: The largest freshwater lake in China. Sci. Rep.-UK.

[41] Wu Z.S., Wang X.L., Chen Y.W., Cai Y.J., Deng J.C. (2018). Assessing river water quality using water quality index in lake taihu basin, China. Sci. Total Environ..

[42] Pesce S.F., Wunderlin D.A. (2000). Use of water quality indices to verify the impact of cordoba city (argentina) on suquia river. Water Res..

[43] Kannel P.R., Lee S., Lee Y.S., Kanel S.R., Khan S.P. (2007). Application of water quality indices and dissolved oxygen as indicators for river water classification and urban impact assessment. Environ. Monit. Assess..

[44] Valentukeviciene M., Bagdziunaite-Litvinaitiene L., Chadysas V., Litvinaitis A. (2018). Evaluating the impacts of integrated pollution on water quality of the trans-boundary neris (viliya) river. Sustainability.

[45] Si yue L.I., Zhang Q.F. (2008). Assessing the water quality in the water source area of the middle route of the south to north water transfer project(danjiangkou reservoir) using a water quality index method. Res. Environ. Sci..

[46] Liu H., Li W. (2011). Dissolved trace elements and heavy metals from the shallow lakes in the middle and lower reaches of the yangtze river region, China. Environ. Earth Sci..

[47] Jun sheng Q.I., Chuan F.U., Huang X.S., Tan J. (2002). Transfer of trace elements in water area ecology systems of the three gorges reservoir. J. Chongqing Univ..

[48] Li F., Huang J.H., Zeng G.M., Yuan X.Z., Li X.D., Liang J., Wang X.Y., Tang X.J., Bai B. (2013). Spatial risk assessment and sources identification of heavy metals in surface sediments from the dongting lake, middle China. J. Geochem. Explor..

[49] Pekey H., Karakas D., Bakoglu M. (2004). Source apportionment of trace metals in surface waters of a polluted stream using multivariate statistical analyses. Mar. Pollut. Bull..

[50] Cotruvo J.A. (2017). 2017 WHO guidelines for drinking water quality: First addendum to the fourth edition. J. Am. Water Works Assoc..

[51] (2019). Epa sets goal of reducing drinking water systems violating health standards. Groundw. Monit. Remediat..

[52] Lee J., Lee S., Yu S., Rhew D. (2016). Relationships between water quality parameters in rivers and lakes: Bod5, cod, nbops, and toc. Environ. Monit. Assess..

[53] Kangabam R.D., Govindaraju M. (2017). Anthropogenic activity-induced water quality degradation in the loktak lake, a ramsar site in the indo-burma biodiversity hotspot. Environ. Technol..

[54] Sánchez E., Colmenarejo M.F., Vicente J., Rubio A., García M.G., Travieso L., Borja R. (2007). Use of the water quality index and dissolved oxygen deficit as simple indicators of watersheds pollution. Ecol. Indic..

[55] Rajendran V., Shrinithivihahshini N.D., Srinivasan B., Rengaraj C., Mariyaselvam S. (2018). Quality assessment of pollution indicators in marine water at critical locations of the gulf of mannar biosphere reserve, tuticorin. Mar. Pollut. Bull..

[56] Yang D., Lin Z., Song W., Chen S., Zhang Y. (2012). Evaluation indexes and methods for water quality in ocean dumping areas. Procedia Environ. Sci..

[57] Avigliano E., Schenone N.F. (2015). Human health risk assessment and environmental distribution of trace elements, glyphosate, fecal coliform and total coliform in atlantic rainforest mountain rivers (south america). Microchem. J..

[58] Liu R., Kang Y., Zhang C., Pei L., Wan S., Jiang S., Liu S., Ren Z., Yang Y. (2014). Chemical fertilizer pollution control using drip fertigation for conservation of water quality in danjiangkou reservoir. Nutr. Cycl. Agroecosyst..

[59] Shen H., Cai Q., Min Z. (2015). Spatial gradient and seasonal variation of trophic status in a large water supply reservoir for the south-to-north water diversion project, China. J. Freshw. Ecol..

[60] Huang J.C., Gao J.F. (2017). An improved ensemble kalman filter for optimizing parameters in a coupled phosphorus model for lowland polders in lake taihu basin, China. Ecol. Model..

[61] Yang Y., Gao B., Hao H., Zhou H., Lu J. (2017). Nitrogen and phosphorus in sediments in China: A national-scale assessment and review. Sci. Total Environ..

[62] Jing H., Xu C., Ridoutt B.G., Wang X., Ren P. (2017). Nitrogen and phosphorus losses and eutrophication potential associated with fertilizer application to cropland in China. J. Clean. Prod..

[63] Filstrup C.T., Downing J.A. (2017). Relationship of chlorophyll to phosphorus and nitrogen in nutrient-rich lakes. Inland Waters.

[64] Karastoyanov V., Bojinov M. (2009). Mechanism of anodic oxidation of tungsten in neutral sulphate-fluoride solutions. J. Solid State Electrochem..

[65] Lu W., Xiang X., Lu Y., Yan X., Xiao L., Liu S. (2017). The temporal-spatial distribution and changes of dissolved oxygen in the changjiang estuary and its adjacent waters for the last 50 a. Acta Oceanol. Sin..

[66] Beale D.J., Karpe A.V., Ahmed W., Cook S., Morrison P.D., Staley C., Sadowsky M.J., Palombo E.A. (2017). A community multi-omics approach towards the assessment of surface water quality in an urban river system. Int. J. Environ. Res. Public Health.

[67] Wu N.C., Schmalz B., Fohrer N. (2012). Development and testing of a phytoplankton index of biotic integrity (p-ibi) for a german lowland river. Ecol. Indic..

[68] Tasdighi A., Arabi M., Osmond D.L. (2017). The relationship between land use and vulnerability to nitrogen and phosphorus pollution in an urban watershed. J. Environ. Qual..

[69] Beutel M.W., Cox S.E., Gebremariam S. (2016). Effects of chironomid density and dissolved oxygen on mercury efflux from profundal lake sediment. Lake Reserv. Manag..

